# Special Issue: Kinase inhibitors

**DOI:** 10.3390/molecules23071818

**Published:** 2018-07-22

**Authors:** Pierre Koch, Stefan Laufer

**Affiliations:** Department of Pharmaceutical and Medicinal Chemistry, Institute of Pharmaceutical Sciences, Eberhard Karls Universität Tübingen, Auf der Morgenstelle 8, 72076 Tübingen, Germany

Protein kinases represent a large and diverse multi-gene family of enzymes, which catalyze the transfer of the γ-phosphate group from its natural co-substrate adenosine triphosphate (ATP) to a free hydroxyl group of an amino acid side chain. They are involved in numerous cell signaling pathways. Diseases might arise when deregulation or mutation of a kinase takes place. Therefore, kinases are promising drug targets for the treatment of several disorders ranging from cancer, autoimmune pathologies, inflammation, or neurodegenerative diseases. After the approval of imatinib in 2001, 38 kinase inhibitors were introduced to the market by the beginning of 2018. Numerous kinase inhibitors are currently in clinical trials.

Structural data—e.g., from the structural genomics consortium (SGC)—as well as the high-quality kinase probe programs from the Chemical Probe Portal and the SGC kept pushing forward both the identification of new kinase targets and the design of novel kinase inhibitors within the last years. The numbers of reported type I½, type III, and type V kinase inhibitors as well as covalent kinase inhibitors have continuously increased in the last few years.

This Special Issue deals with the design and development of novel inhibitors addressing different kinases as well as with strategies to inhibit kinases. The Special Issue consists of eight original research articles and four reviews, as briefed below.

Brenner et al. described the efficacy of six inhibitors of CDC25, dual-specificity phosphatases that activate cyclin-dependent kinases, on acute myeloid leukemia cells isolated from patients [1]. The responders to CDC25 inhibition were identified by analysis of global gene expression profiles which were then compared to the ones of non-responders. 

In their study, Halekotte et al. designed and synthesized optimized 4,5-diarylimidazoles as ATP-competitive inhibitors of CK1δ and discussed their structural relation to the p38α mitogen-activated protein (MAP) kinase [2]. The results obtained by SAR analysis were supported by X-ray structures of both CK1δ and p38α MAP kinase crystals incubated with different inhibitors as well as by molecular modeling studies. The most potent inhibitors of their series are among the most potent CK1δ-targeting agents published to date. 

Zhang et al. described the ligand-based design of pyrrolo[2,3-*b*]pyrazine-based FGFR inhibitors starting from a c-Met inhibitor [3]. Extensive SAR-elaboration to enhance the binding efficacy for this novel class of FGFR inhibitors were reported.

An analysis of kinase inhibitors and their specificity with respect to their scaffold diversity is described by Dimova and Bajorath [4]. 

Yang et al. reported the synthesis of novel pyrrolidone-fused methylpyrrole derivatives and their biological evaluation as potential multi-target tyrosine kinase receptor inhibitors [5]. An extensive evaluation of this class of compounds in different assay is reported. Molecular docking studies based on recently published X-ray structures of VEGFR-2 supported the results obtained by SAR analysis and revealed the binding mode for their class of compounds to be similar to one of the approved inhibitor sunitinib. 

In their study, Tegethoff et al. showed that 1-methylisoindigo is a Stat3-related inhibitor of various tyrosine kinases [6]. The authors synthesized a small library of 1-methylisoindigo derivatives and compared their anti-proliferative effects on tumor cell lines. Accessible positions on bis-indole backbone for further chemical modification were identified. 

The metabolic stability of two potent pyridinylimidazole-based p38α mitogen-activated protein (MAP) kinase inhibitors ML3403 and LN950 was improved by Heider et al. [7]. The novel metabolically stable inhibitors were extensively evaluated in various assays and compared to their parent compounds. Exchange of their metabolic hot spot resulted in analogs showing a higher binding affinity towards the target kinase than the parent compounds. 

The number of both approved kinase inhibitors and kinase inhibitors in clinical trials increases continuously. In their research article, Carles et al. presented an extensive analysis of their monthly-updated Protein Kinase Inhibitor Database (PKID) [8]. The PKID contains a lot of information for more than 180 kinase inhibitors that received an international nonproprietary name.

In their review, Lee at al. summarized recent advances in the targeting of the p38α MAP kinase as a potential strategy for the treatment of Alzheimer’s disease [9]. Inhibitors of this serine/threonine kinase offering neuroprotection as well as their limitation for the treatment of Alzheimer’s disease were discussed.

Lee et al. discuss the pro- and anti-tumorigenesis functions of senescence as well as the roles of kinase modulators in these processes [10]. An overview of FDA-approved kinase modulators and their influences on senescence regulation is provided.

In their comprehensive review, Cheng et al. summarized the recent progress of MEK inhibitors and focused on marketed MEK inhibitors and inhibitors in clinical trials [11]. Clinical and preclinical study results of MEK inhibitors were presented and combinations of MEK inhibitors with other therapies were summarized. 

Schmidt et al. presented a timely review on the two members of the WEE kinase family, WEE1 and PKMYT1 [12]. Both kinases play pivotal roles in the DNA-damage recovery. This review features structural analysis of WEE family kinases and discussed WEE1 and PKMYT1 as potential drug targets in cancer therapy.

## References

[1] Brenner A.K., Reikvam H., Rye K.P., Hagen K.M., Lavecchia A., Bruserud O. (2017). CDC25 Inhibition in Acute Myeloid Leukemia-A Study of Patient Heterogeneity and the Effects of Different Inhibitors. Molecules.

[2] Halekotte J., Witt L., Ianes C., Kruger M., Buhrmann M., Rauh D., Pichlo C., Brunstein E., Luxenburger A., Baumann U. (2017). Optimized 4,5-Diarylimidazoles as Potent/Selective Inhibitors of Protein Kinase CK1 delta and Their Structural Relation to p38 alpha MAPK. Molecules.

[3] Zhang Y., Liu H.C., Zhang Z., Wang R.F., Liu T.C., Wang C.Y., Ma Y.C., Ai J., Zhao D.M., Shen J.K. (2017). Discovery and Biological Evaluation of a Series of Pyrrolo[2,3-b]pyrazines as Novel FGFR Inhibitors. Molecules.

[4] Dimova D., Bajorath J. (2017). Assessing Scaffold Diversity of Kinase Inhibitors Using Alternative Scaffold Concepts and Estimating the Scaffold Hopping Potential for Different Kinases. Molecules.

[5] Yang T.H., Lee C.I., Huang W.H., Lee A.R. (2017). Synthesis and Evaluation of Novel 2-Pyrrolidone-Fused (2-Oxoindolin-3-ylidene)methylpyrrole Derivatives as Potential Multi-Target Tyrosine Kinase Receptor Inhibitors. Molecules.

[6] Tegethoff J., Bischoff R., Saleh S., Blagojevic B., Merz K.H., Cheng X.L. (2017). Methylisoindigo and Its Bromo-Derivatives Are Selective Tyrosine Kinase Inhibitors, Repressing Cellular Stat3 Activity, and Target CD133+ Cancer Stem Cells in PDAC. Molecules.

[7] Heider F., Haun U., Doring E., Kudolo M., Sessler C., Albrecht W., Laufer S., Koch P. (2017). From 2-Alkylsulfanylimidazoles to 2-Alkylimidazoles: An Approach towards Metabolically More Stable p38 alpha MAP Kinase Inhibitors. Molecules.

[8] Carles F., Bourg S., Meyer C., Bonnet P. (2018). PKIDB: A Curated, Annotated and Updated Database of Protein Kinase Inhibitors in Clinical Trials. Molecules.

[9] Lee J.K., Kim N.J. (2017). Recent Advances in the Inhibition of p38 MAPK as a Potential Strategy for the Treatment of Alzheimer’s Disease. Molecules.

[10] Lee C.S., Baek J., Han S.Y. (2017). The Role of Kinase Modulators in Cellular Senescence for Use in Cancer Treatment. Molecules.

[11] Cheng Y., Tian H.Q. (2017). Current Development Status of MEK Inhibitors. Molecules.

[12] Schmidt M., Rohe A., Platzer C., Najjar A., Erdmann F., Sippl W. (2017). Regulation of G2/M Transition by Inhibition of WEE1 and PKMYT1 Kinases. Molecules.

